# Association of MicroRNA Expression and Serum Neurofilament Light Chain Levels with Clinical and Radiological Findings in Multiple Sclerosis

**DOI:** 10.3390/ijms251810012

**Published:** 2024-09-17

**Authors:** María Inmaculada Domínguez-Mozo, Ignacio Casanova, Enric Monreal, Lucienne Costa-Frossard, Susana Sainz-de-la-Maza, Raquel Sainz-Amo, Yolanda Aladro-Benito, Pedro Lopez-Ruiz, Laura De-Torres, Sara Abellán, Maria Angel Garcia-Martinez, David De-la-Cuesta, Daniel Lourido, Angel Torrado-Carvajal, Carol Gomez-Barbosa, Carla Linares-Villavicencio, Luisa Maria Villar, Carlos López-De-Silanes, Rafael Arroyo, Roberto Alvarez-Lafuente

**Affiliations:** 1 Research Group in Environmental Factors of Neurodegenerative Diseases, Instituto de Investigación Sanitaria del Hospital Clínico San Carlos (IdISSC), Red de Enfermedades Inflamatorias (REI), 28040 Madrid, Spain; garcia.angel23@gmail.com (M.A.G.-M.); ddelacue@ucm.es (D.D.-l.-C.); ralvarezlafuente@yahoo.es (R.A.-L.); 2 Department of Neurology, Hospital Universitario de Torrejón, 28850 Madrid, Spain; i.casanovap@gmail.com (I.C.); lauravdetorres@gmail.com (L.D.-T.); sabellan@torrejonsalud.com (S.A.); carlosdesilanes@gmail.com (C.L.-D.-S.); 3 School of Medicine, Universidad Francisco de Vitoria, 28223 Madrid, Spain; 4 Department of Neurology, Hospital Universitario QuironSalud Madrid, Pozuelo de Alarcón, 28223 Madrid, Spain; pedro.lopez.ruiz@tutanota.com (P.L.-R.); rafaelarroyo09@gmail.com (R.A.); 5 Department of Neurology, Hospital Universitario Ramón y Cajal, Red de Enfermedades Inflamatorias (REI), Instituto Ramón y Cajal de Investigación Sanitaria, Universidad de Alcalá, 28034 Madrid, Spain; enricmonreal@outlook.com (E.M.); lufrossard@yahoo.es (L.C.-F.); susanasmc85@hotmail.com (S.S.-d.-l.-M.); raquelsainzamo@gmail.com (R.S.-A.); 6 Department of Neurology, Hospital Universitario de Getafe, 28905 Madrid, Spain; yolanda.aladro@salud.madrid.org; 7 Department of Radiology, Hospital Universitario Ramón y Cajal, Instituto Ramón y Cajal de Investigación Sanitaria, Universidad de Alcalá, 28034 Madrid, Spain; louridodaniel@hotmail.com; 8 Medical Image Analysis and Biometry Laboratory, Universidad Rey Juan Carlos, Móstoles, 28933 Madrid, Spain; angel.torrado@urjc.es; 9 Department of Radiology, Hospital Universitario de Torrejón, 28850 Madrid, Spain; cfgomez@torrejonsalud.com (C.G.-B.); cglinares@torrejonsalud.com (C.L.-V.); 10 Department of Immunology, Hospital Universitario Ramón y Cajal, Red de Enfermedades Inflamatorias (REI), Instituto Ramón y Cajal de Investigación Sanitaria, Universidad de Alcalá, 28034 Madrid, Spain; luisamaria.villar@salud.madrid.org

**Keywords:** multiple sclerosis, microRNAs (MiRNAs), neurofilament light (NfL), magnetic resonance imaging (MRI), processing speed test (PST)

## Abstract

microRNAs (miRNAs) are promising biomarkers for many diseases, including multiple sclerosis (MS). The neurofilament light chain (NfL) is a biomarker that can detect axonal damage in different neurological diseases. The objective of this study was to evaluate the association of the expression profile of pre-selected miRNAs and NfL levels with clinical and radiological variables in MS patients. We conducted a 1-year longitudinal prospective study in MS patients with different clinical forms. We measured clinical disability using the expanded disability status scale (EDSS), the magnetic resonance imaging (MRI) volumetry baseline, and cognitive functioning using the processing speed test (PST) at baseline and 1 year later. Selected serum miRNAs and serum NfL (sNfL) levels were quantified. Seventy-three patients were recruited. MiR-126.3p correlated with EDSS and cognitive status at baseline and miR-126.3p and miR-9p correlated with cognitive deterioration at 1 year. Correlations with regional brain volumes were observed between miR-126.3p and the cortical gray matter, cerebellum, putamen, and pallidum; miR-146a.5p with the cerebellum and pallidum; miR-29b.3p with white matter and the pallidum; miR-138.5p with the pallidum; and miR-9.5p with the thalamus. sNfL was correlated with miR-9.5p. miR-146a.5p was also associated with the MS phenotype. These data justify future studies to further explore the utility of miRNAs (mirR-126.3p, miR-146.5p, and miR.9-5p) and sNfL levels as biomarkers of MS.

## 1. Introduction

MicroRNAs (miRNAs) are a type of small non-coding interfering RNAs that regulate gene activity at a post-transcriptional level, mainly by reducing or inhibiting the translation of complementary mRNA targets [1,2]. They participate in almost every cellular process, and their expression can be modified in different physiological and pathological scenarios. For these reasons, they are promising biomarkers in many diseases [3,4,5,6,7]. Several studies have demonstrated the potential role of miRNAs in multiple sclerosis (MS) [8,9,10,11,12,13,14,15,16]. However, there is still a need to further improve our knowledge of these molecules in MS, and establish their possible applications, such as differentiating MS phenotypes, predicting the risk of relapse, for prognosis of the therapeutic response to disease-modifying drugs, and even deciphering new targets for the development of novel treatments.

In contrast, the neurofilament light chain (NfL) is a biomarker that can detect axonal damage in many neurological diseases. Specifically, in MS, the NfL has been associated with inflammation [17,18] and the risk of disability in the short and long term [18,19,20], and is used as a tool to monitor clinical evolution and treatment response [21,22].

The objective of this study was to further investigate the potential utility of a set of specific microRNAs previously identified as biomarkers in MS [16,23] in a larger cohort of MS patients and to combine these data with the more studied and validated serum NfL (sNfL).

## 2. Results

For our cohort, 73 patients (48 females) were recruited: 6 with clinically isolated syndrome (CIS), 49 with relapsing-remitting MS (RRMS), 7 with secondary progressive MS (SPMS), 6 with primary progressive MS (PPMS), and 5 with radiologically isolated syndrome (RIS). The mean age at MS onset was 35.3 y/o (SD: 10.6), the mean age at study was 39.5 y/o (SD: 10.6), and the mean expanded disability status scale (EDSS) was 2.0 (SD: 1.4). The demographic and clinical data of the patients are summarized in Table 1.

EDSS was inversely correlated with processing speed test (PST). Both EDSS and PST were also correlated with every magnetic resonance imaging (MRI) volumetry assessment except for pallidum atrophy (Table 2).

In univariate analysis, miR-126.3p was associated with EDSS, PST, and PST change at 1 year, while miR-9.5p was associated with PST change (Table 3).

Regarding the MRI data, several miRNAs correlated with different brain atrophy measurements (Table 4).

The sNfL level did not achieve statistical significance for any clinical or radiological variable, but it was correlated with miRNA-9.5p (Table 3).

Those variables with correlations with clinical or MRI data were further analyzed with E, including sex, age, MS duration, and MS phenotype as independent variables. miR-9.5p maintained a statistical association with PST-change (beta = 0.04; *p* = 0.007). miR-126.3p kept a statistical association with EDSS (beta = 1.07; *p* = 0.008; also associated with the MS phenotype), PST-change (beta = −0.131; *p* = 0.031), cerebellum volume (beta = −22.4; *p* = 0.037), and putamen volume (beta = −2.1; *p* = 0.017; also associated with age at study). In addition, miR-146a.5p was associated with cerebellum volume (beta = 12.72; *p* < 0.0001, also associated with sex and MS phenotype), and pallidum volume (beta = −0.39; *p* < 0.0001; also associated with age) (Table 5).

**Figure 1:**
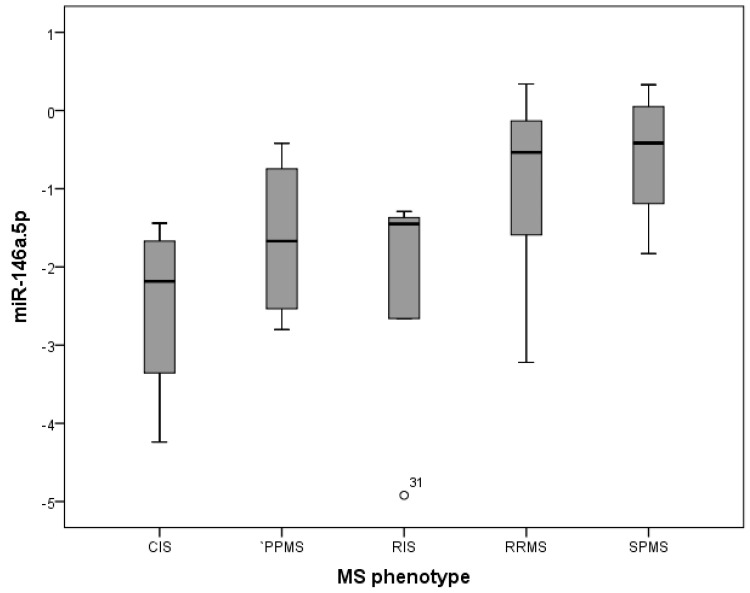
Association between miR-146a.5p and clinical multiple sclerosis phenotypes. CIS: Clinically isolated syndrome; PPMS: primary progressive multiple sclerosis; RIS: radiologically isolated syndrome; RRMS: relapsing-remitting multiple sclerosis; SPMS: secondary progressive multiple sclerosis.

Finally, miR-146a.5p was also associated with the clinical MS phenotype, with median values increasing in this order: CIS, PPMS, RIS, RRMS, and SPMS (Kruskal–Wallis, *p* = 0.012) (Figure 1). The miR-146a.5p levels were statistically different (Mann–Whitney U) between CIS and RRMS (*p* = 0.019), CIS and SPMS (*p* = 0.23), and RIS and RRMS (*p* = 0.023); additionally, we found a tendency between RIS and SPMS (*p* = 0.062) and between SPMS and PPMS (*p* = 0.089).

## 3. Discussion

This study is the continuation of previous work, including a bigger sample [1,2]. After the addition of more recent patients, we were able to replicate some of the previous associations between clinical and/or radiological variables with several miRNAs. In the same way, the bigger sample allowed us to perform multivariate analysis, with some of these associations maintaining statistical significance. Most of these associations were also in line with our previous results and with others reported in the literature [1,2,3,4]. All these data reinforce the value of our findings. Besides this, the clinical, radiological, and epidemiological composition of our study clearly resembles the main characteristics of the MS population. As expected, we found a strong and repeated association between EDSS and PST, and also between these two tests and all the radiological variables described in Table 2 [5,6,7]. These findings further strengthen the previously published results. Finally, the inclusion of new patients, the routine clinical settings of the study, and small differences in methodology between our different studies, with different MRI data and analysis software, also give strong external validity to our findings.

miR-126.3 presented the most relevant results in this study, demonstrating simple correlations with almost every clinical and radiological variable (a positive correlation with EDSS, and a negative correlation with PST, PST-change, CGMV, and the cerebellum, putamen, and pallidum volume), with most of them also being statistically significant after multivariate analysis (EDSS, PST-change, cerebellum, and putamen). These yielded similar results compared to previous studies [1,2], with a consistent direction of association across all these clinical and radiological variables. There are several studies connecting this microRNA to MS, with mixed results. Some of them suggest that it could have an anti-inflammatory effect [8], while others find higher levels of this molecule with higher disease activity and a reduction after treatment with natalizumab [9]. Our results reinforce the data for the pathogenic effect of this microRNA over the course of MS. At this point, it would not be possible for us to propose a hypothesis for the mechanisms behind this effect. miR-126 has been implicated in immune system regulation [10], but it is mainly an endothelial-specific microRNA [11], and its functions could also be related to angiogenesis, leukocyte trafficking, and blood-brain barrier integrity [12,13,14]. We tried to investigate this possible mechanism of action of mir-126.3p by studying its relationship with gadolinium sequences, but only two patients had contrast enhancement lesions, so we could not corroborate this hypothesis at this point.

For miR-146a.5p, we found a strong correlation with the cerebellum and pallidum volume, which also surpassed multivariate analysis in both cases (see the results included in Table 4 and Table 5). These results are somehow confusing, as they have opposite signs of association (positive for cerebellum volume and negative for pallidum volume). Additionally, we also could not find any correlations with clinical variables (EDSS or cognitive dysfunction) that were present in previous studies [15].

However, interestingly, we found a correlation between miR-146a.5p median values and the MS phenotype, with an increase in the following order: CIS, PPMS, RIS, RRMS, and the highest values in SPMS. Differences in microRNA profiles across MS phenotypes have previously been published for other microRNAs [16,17,18], but not for miR-146a.5p. With these results, we think that miR-146a.5p could, thus, be related to the higher inflammatory activity of MS (higher in RRMS than in CIS—which, according to the latest criteria, is a very infrequent phenotype with very little inflammation) and also to higher neuronal damage or less neuroprotection (highest values in SPMS). It is known that this microRNA exerts many anti-inflammatory effects [19] and facilitates oligodendrocyte differentiation [20,21], so their higher values could be a compensatory mechanism to try to counterbalance these pathogenic damages.

The lower values of miR-146a.5p in PPMS compared to SPMS could explain the lack of association with EDSS in this study, as both phenotypes had similar EDSS. In the same way, the similar values of miR-146a.5p in RIS and PPMS and lower values to other phenotypes of MS (excluding CIS) would suggest that RIS could be more closely related to progressive forms of the disease rather than benign MS or the prodromal phase of relapsing MS. This hypothesis is also supported by the epidemiological data (older age for RIS compared to CIS), clinical data (lower PST values in RIS than in CIS), and other lab results (higher sNfL values in RIS than in CIS) (Table 2). Finally, these low values, similar in RIS and PPMS without increasing their levels as disability progresses in PPMS (as opposed to the highest values in SPMS) could reflect slight differences in some mechanisms of progression in PPMS compared to SPMS. Most experts agree that MS is a continuum with overall shared and overlapping mechanisms between these different phenotypes [22], but there could be some differences [23]. In this regard, it has been demonstrated that microglia are the major source of miR-146a.5p in the central nervous system, and this microRNA could play an important role in the gene regulation of active MS lesions [24], which are regarded as a key mechanism of progression in SPMS [25].

miR-138.3p is a potent tumor suppressor with different and opposing mechanisms [26,27,28]. We also obtained different results in our previous studies, with a positive correlation with pallidum and amygdala volumes [1] but a negative association with No Evidence of Disease Activity of −3 (no relapses, no disability progression, and no MRI activity; NEDA3) [2]. In this work, we again found a simple correlation, albeit not in multivariate analysis, with pallidum volume, which would reinforce its possible neuroprotective and remyelinating effects [29].

miR-29b is an inflammatory molecule upregulated in the CD4 lymphocytes of MS patients that promotes Th1 responses [30]. The higher values found in association with poorer measurements of white matter and pallidum volume would be in line with these data, as well as the reduction of its levels after some disease-modifying drugs [30,31].

Finally, we could not find any association between sNfL and clinical or radiological variables. sNfL is a validated biomarker of axonal damage, progression, and prognosis in MS [32,33,34,35,36], mainly related to inflammation [37]. In our sample, the mixing of progressive and relapsing MS phenotypes with different pathological mechanisms of progression, and not only inflammation, could explain this lack of association. Conversely, we found an interesting positive correlation between sNfL and miRNA-9.5p. This is another pro-inflammatory microRNA [38,39] that has been linked with the pathogenic mechanisms of MS in several studies and animal models [40,41]. In this work, we found a correlation of miR-9.5p with thalamus volume in simple analysis, similar to that seen in our previous results. In contrast, when performing both univariate and multivariate analysis, we observed an association of miR-9.5p with PST-improve, thus suggesting a protective role of miR-9.5p. We would need to conduct new studies to clarify this point and confirm the relationship of miR-9.5p with MS and its true effects.

Regarding this issue of the biological and physiological characterization of the miRNAs included in this study, there is little information in the literature at this moment. Only for miR-146a-5p and miR-29b, there are studies describing their possible targets [42,43,44]

We are aware of some limitations of this study. First of all, there are some statistical issues and numerical imbalances between groups. Secondly, the low number of gadolinium-enhancing lesions, the unavailability of other interesting MRI data such as on T2 and T1 lesion volumes, and the lack of follow-up MRI and EDSS assessments. We aim to continue with these studies of microRNAs in the future, incorporating these analyses.

## 4. Materials and Methods

### 4.1. Study Design

The present study was designed as a longitudinal prospective analytic study in a cohort of MS patients attending the demyelinating diseases unit at the University Hospital Torrejón, University Hospital Ramón y Cajal, and University Hospital Getafe, Madrid, Spain. During the first visit, we measured the EDSS, PST, MRI volumetry, microRNAs, and sNfL levels. PST was repeated once during the second visit, conducted 1 year later, to evaluate cognitive decline.

We selected patients with a diagnosis of clinically isolated syndrome (CIS), relapsing-remitting (RRMS), secondary progressive (SPMS), and primary progressive (PPMS) multiple sclerosis according to McDonald in 2017 [24], and radiologically isolated syndrome (RIS) according to Okuda 2009 [25], without active treatment (naïve patients or at least 6 months without any disease-modifying treatment).

Exclusion criteria included: relapse or corticosteroid treatment in the 3 months previous to the study, and any contraindication for performing an MRI.

Sex, age at disease onset, and clinical MS phenotype according to Lublin (2013) [26] were collected through a medical chart review during clinic visits. Clinical disability was measured with the EDSS. Cognitive function was determined with the PST [27]. PST raw values were transformed into percentiles and adjusted according to age, sex, and academic level. Cognitive evolution at 1 year was calculated using the relative PST change (final PST–basal PST, divided by basal PST).

All patients provided their consent to participate in the study. The study complied with the Helsinki Declaration [28] and was approved by the ethical committee of the Hospital Universitario Fundación Jiménez Diaz (Madrid, Spain).

### 4.2. MicroRNA Selection and Serum Neurofilament Light Chain Analysis

Peripheral blood samples were collected from each enrolled patient in red-top vacutainer tubes without additives during the first visit (BD Vacutainer^®^, Franklin Lakes, NJ, USA), centrifuged at 920× *g* for 15 min at room temperature to separate serum, and stored at −80 °C until RNA extraction. Previous to nucleic acid purification, the serum was thawed at room temperature. To remove cryoprecipitates, 300 μL samples of thawed serum were centrifuged for 5 min at 3000× *g* and 4 °C, and 200 μL of the supernatant was transferred to a new tube. Cell-free total RNA was extracted using the miRNeasy Serum/PlasmaAdvanced Kit (Qiagen, Hilden, Germany) according to the manufacturer’s protocol. During the RNA extraction process, the UniSp2, UniSp4, and UniSp5 RNA Spike-in mix (RNA Spike-in Kit for RT, Qiagen^®^, Germantown, MD, USA) was added to provide a control for the quality of RNA isolation. The total RNA was reverse-transcribed using the miRCURY LNA RNA kit (Qiagen, Hilden, Germany), following the manufacturer’s instructions, to generate universal cDNA templates for all miRNAs present in the sample. The synthetic UniSp6 RNA spike-in mix (Qiagen, Hilden, Germany) was added to each sample during this process to provide a control for the quality of the cDNA synthesis; the reaction was performed in the VeritiTM thermal cycle (Applied BiosystemsTM, Waltham, MA, USA). Prepared complementary DNA samples were stored at −20 °C until use. The miRCURY LNA miRNA QC PCR panel test (Qiagen, Hilden, Germany) was performed to analyze the robustness of the RNA isolation process and the quality of isolated miRNA. The panel contains matching locked nucleic acid (LNA) PCR assays for the detection of: the RNA spike-in mixes (UniSp2, UniSp4, and UniSp5); the spike-ins UniSp6 and cel-miR-39-3p (not added in our experiments), to monitor cDNA synthesis; theUniSp3 IPC (inter-plate calibrator) to check if the qPCR was successful; four potential endogenous miRNAs: miR-103-3p, miR-191-5p, miR-30c-5p, and miR- 124-3p, along with miR-451a and miR-23a-3p, which serve as hemolysis markers. MiRNA-specific quantification was performed using the miRCURY LNA SYBR green kit (Qiagen, Hilden, Germany) according to the manufacturer’s instructions in a LightCycler 96 instrument (Roche Applied Science, Basel, Switzerland). The miRCURY LNA miRNA Custom PCR panels were performed using only the samples with successful results in the miRCURY LNA miRNA QC PCR panel.

A total of 8 miRNAs where the expression profile was found to be statistically associated with clinical disability and brain atrophy in previous works (miR-9.5p, miR-29.3p, miR-34a.5p, miR-126.3p, miR-138.5p, miR146a.5p, miR-200c.3p, and miR-223.3p) [16,23] were included in the miRCURY LNA miRNA Custom PCR panels, apart from the four potential endogenous miRNAs and the spike-ins UniSp6 and UniSp3. MiRNA-specific quantification was performed using the miRCURY LNA SYBR green kit (Qiagen, Hilden, Germany) according to the manufacturer’s instructions in a LightCycler 96 instrument (Roche Applied Science). All samples were run as duplicates. Normalization was performed using the mean expression of two endogenous miRNAs: miR191-5p and miR30c-5p. The normalized cycle quantification (Cq) value was calculated as the mean Cq—endogenous Cq.

sNfL was measured with an SR-X instrument (Quanterix, Lexington, MA, USA) using the single molecule array NF-light Advantage kit technique (Quanterix, Billerica, MA, USA) (Manouchehrinia A et al.). Raw sNfL values were transformed to Z-scores [29,30]. Based on previous studies, a cutoff value for a high sNfL Z-score was established at 1.5.

### 4.3. MRI and Brain Volume Analysis

MRI images were acquired following the magnetic resonance imaging in multiple sclerosis (MAGNIMS) recommendations on the use of brain MRI scans in multiple sclerosis [31], with a minimum magnetic field strength of 1.5T, a maximum slice thickness of 3 mm without a gap, and the following sequences: axial T1-weighted pre- and post-gadolinium, axial T2-weighted and/or proton density, and axial and sagittal T2-fluid-attenuated inversion recovery (FLAIR). An isovolumetric sagittal T1 (3D-SPGR) sequence was performed with the following parameters TR = 8.5 ms; TE = 3.2 ms; TI = 700 ms; flip angle (FA) = 12; bandwidth = 31.25 kHz; and a highest voxel size of 1 × 1 × 1 mm to perform the volumetric analysis. Whole brain, cortical grey matter, white matter, cerebellum, thalamus, caudate, pallidum, and putamen volumes were computed using the automated mdbrain brain volumetry module^®^ and NeuroQuant^®^ MS software.

### 4.4. Statistics

The Statistical Package for Social Sciences, version 19.0 (IBM SPSS, Inc., Chicago, IL, USA) was used for the statistical analyses. We described numerical variables, which were expressed as median and interquartile ranges, and categorical variables were expressed as percentages. Associations between miRNAs and sNfL with clinical outcomes (EDSS, basal PST, and PST-change) and MRI data were studied using the Spearman correlation (r_s_). Those variables with statistical significance were further analyzed through multivariate linear regression analysis, using the corresponding miRNA/sNfL as the independent variable. We also included sex, age at study, MS duration, and MS phenotype as the other independent variables for every model, with EDSS and MRI as the dependent variables; MS duration and MS phenotype acted as the other independent variables for PST analysis, as raw values of PST are automatically transformed to Z-scores that are adjusted for sex, age and education. Statistical significance was set at *p* ≤ 0.05.

## 5. Conclusions

In conclusion, our results add more data in the field of studying microRNAs as possible biomarkers of prognosis in MS or to monitor disease progression or treatment response. They could also be valuable tools in advancing our knowledge of MS pathophysiology regarding both the inflammatory and neurodegenerative processes driving this disease, thereby helping in deciphering and designing new therapeutic targets.

We would like to highlight miR-126.3p, since the present study provides longitudinal evidence in support of a prognostic role concerning processing speed (PST), a cognitive domain frequently impaired throughout the course of MS. Moreover, the clinical–radiological correlations for miR-126.3p, miR-146.5p, and miR-9.5p are in line with the consistent associations repeatedly found across studies, suggesting a relevant role for different miRNAs in MS pathogenesis.

## Figures and Tables

**Table 1:** Demographic and clinical data.

	Sex N (F:M)	Age Diagnosis Mean (±SD) (Years)	Age Study Mean (±SD) (Years)	EDSS Mean (±SD) Median (P25, P75)	PST (Percentiles) Mean (±SD)	sNfL Mean (±SD) (pg/mL)
All patients	73 (48:25)	35.3 (10.6)	39.5 (10.6)	2.0 (1.4) 2.0 (1.0, 3.0)	49.3 (29.7)	12.3 (7.3)
RIS	5 (4:1)	44.2 (4.6)	45.9 (3.3)	1.4 (1.1) 1.0 (1.0, 2.5)	50.9 (29.1)	13.3 (11.4)
CIS	6 (4:2)	37.4 (9.6)	37.6 (9.5)	1.4 (0.4) 1.5 (1.0, 1.5)	65.3 (25.3)	5.8 (2.4)
RRMS	49 (34:15)	32.8 (10.4)	37.1 (10.5)	1.7 (1.3) 1.5 (0, 2.5)	53.8 (29.1)	13.7 (7.5)
SPMS	7 (4:3)	41.5 (10.6)	51.6 (7.8)	3.7 (1.5) 4.0 (2.5, 5.0)	12.9 (13.9)	13.5 (8.2)
PPMS	6 (2:4)	38.8 (11.5)	41.7 (9.1)	3.3 (0.9) 3.0 (2.5, 4.0)	32.3 (20.3)	9.5 (1.4)

EDSS: Expanded disability status scale; PST: processing speed test. sNfL: Serum neurofilament light chain; N: number of patients. F: Female; M: male. P25: Percentile 25; P75: percentile 75; SD: standard deviation. RIS: Radiologically isolated syndrome; CIS: clinically isolated syndrome; RRMS: relapsing-remitting multiple sclerosis; SPMS: secondary progressive multiple sclerosis; PPMS: primary progressive multiple sclerosis.

**Table 2:** Correlations between clinical and brain volume data.

	PST r_s_; *p*	WBV r_s_; *p*	WMV r_s_; *p*	CGMV r_s_; *p*	Cerebellum r_s_; *p*	Caudate r_s_; *p*	Putamen r_s_; *p*	Pallidum r_s_; *p*	Thalamus r_s_; *p*
EDSS	* −0.66; < 0.001	* −0.36; 0.026	* −0.36; 0.026	* −0,35; 0.03	* −0,53; <0.001	* −0.48; 0.002	* −0.53; <0.001	0.24; 0.14	* −0.44; 0.005
PST	-	* 0.41; 0.011	* 0.34; 0.038	* 0.44; 0.006	* 0.48; 0.002	* 0.24; 0.16	* 0.51; 0.001	−0.17; 0.31	* 0.47; 0.003

EDSS: Expanded disability status scale. PST: Processing speed test. WBV: Whole brain volume; WMV: white matter volume; CGMV: cortical grey matter volume; r_s_: Spearman rank correlation coefficient. * *p* < 0.05.

**Table 3:** Correlations between microRNAs and Z-sNfL, with clinical data.

	EDSS r_s_; *p*	PST r_s_; *p*	PST-Change r_s_; *p*	Z-sNfL r_s_; *p*
miR-9.5p	0.17; 0.33	0.03; 0.87	* 0.57; 0.041	* 0.53; 0.043
miR-29b.3p	0.06; 0.65	0.02; 0.89	0.33; 0.11	0.34; 0.058
miR-34a.5p	0.11; 0.42	0.1; 0.46	−0.25; 0.26	−0.04; 0.84
miR-126.3p	* 0.3; 0.019	* −0.28; 0.028	* −0.41; 0.04	−0.33; 0.067
miR-138.5p	0.38; 0.1	−0.3; 0.22	−0.68; 0.09	0.14; 0.76
miR-146a.5p	−0.11; 0.39	0.17; 0.19	0.06; 0.78	0.28; 0.12
miR-200c.3p	0.08; 0.52	−0.01; 0.96	0.35; 0.08	0.26; 0.15
miR-223.3p	0.05; 0.71	0.15; 0.24	0.24; 0.26	0.24; 0.18
Z-sNfL	0.32; 0.85	−0.17; 0.32	0.26; 0.27	-

EDSS: Expanded disability status scale; PST: processing speed test; Z-sNfL: serum neurofilament light chain Z-score; r_s_: Spearman rank correlation coefficient. * *p* < 0.05.

**Table 4:** Correlations between microRNAs and sNfL, with MRI data.

	WBV r_s_; *p*	WMV r_s_; *p*	CGMV r_s_; *p*	Cerebellum r_s_; *p*	Caudate r_s_; *p*	Putamen r_s_; *p*	Pallidum r_s_; *p*	Thalamus r_s_; *p*
miR-9.5p	−0.24; 0.27	−0.29; 0.17	−0.08; 0.69	0.06; 0.78	−012; 0.58	−0.08; 0.72	−0.36; 0.088	* −0.44; 0.03
miR-29b.3p	−0.16; 0.35	* −0.33; 0.05	−0.01; 0.97	0.2; 0.23	−0.09; 0.6	−0.04; 0.82	* −0.35; 0.034	−0.28; 0.088
miR-34a.5p	−0.16; 0.39	−0.24; 0.2	−0.03; 0.86	0.12; 0.51	0.15; 0.42	−0.09; 0.6	−0.24; 0.2	−0.15; 0.41
miR-126.3p	−0.17; 0.32	−0.02; 0.92	* −0.34; 0.043	* −0.6; <0.0001	0.02; 0.91	* −0.48; 0.003	* 0.39; 0.017	−0.13; 0.44
miR-138.5p	0.04; 0.89	−0.04; 0.91	0.28; 0.38	−0.13; 0.7	0.32; 0.31	0.05; 0.88	* 0.72; 0.008	0.19; 0.56
miR-146a.5p	−0.08; 0.62	−0.25; 0.13	0.07; 0.7	* 0.58; <0.001	−0.17; 0.33	0.25; 0.14	* −0.64; <0.001	−0.26; 0.12
miR-200c.3p	−0.12; 0.5	−0.11; 0.52	−0.04; 0.63	0.08; 0.65	−0.11; 0.54	0.1; 0.56	−0.31; 0.068	−0.17; 0.34
miR-223.3p	−0.13; 0.43	−0.01; 0.95	−0.15; 0.37	0.19; 0.25	0.003; 0.99	0.12; 0.47	0.19; 0.27	0.11; 0.52
Z-sNfL	−0.16; 0.57	−0.14; 0.63	0.18; 0.52	0.09; 0.75	0.26; 0.36	−0.001; 0.9	−0.12; 0.67	−0.29; 0.29

WBV: Whole brain volume; WMV: white matter volume; CGMV: cortical grey matter volume. Z-sNfL: Serum neurofilament light chain Z-score. r_s_: Spearman rank correlation coefficient. * *p* < 0.05.

**Table 5:** Multivariate regression analysis.

MiRNA	Spearman Correlation		Multivariate Regression	
	Clinical/MRI	r_s_; *p*	Clinical/MRI	beta; *p*
miR-9.5p	Thalamus PST-change	r_s_ = −0.44; 0.03 r_s_ = 0.571; 0.041	Thalamus PST-change	*p* = 0.207 * b = 0.04; 0.007
miR-29b	WMV Pallidum	r_s_ = −0.33; 0.05 r_s_ = −0.35; 0.034	WMT Pallidum	*p* = 0.084 *p* = 0.23
miR-126.3p	EDSS PST PST-change CGMV Cerebellum Putamen Pallidum	r_s_ = 0.29, 0.019 r_s_ = −0.28; 0.028 r_s_ = 0.414; 0.04 r_s_ = −0.34; 0.043 r_s_ = −0.6; <0.0001 r_s_ = −0.45; 0.003 r_s_ = −0.39; 0.017	EDSS PST PST-change CGMV Cerebellum Putamen Pallidum	* b = 1.07; 0.008 (and RRMS/SPMS-PPMS) *p* = 0.127 (SPMS-PPMS) * b = −0.131; 0.031 *p* = 0.256 (sex) * b = −22.4; 0.037 * b = −2.1; 0.017 (and age) *p* = 0.362
miR-138.3p	Pallidum	r_s_ = 0.72; 0.008	Pallidum	*p* = 0.173
miR-146a.5p	Cerebellum Pallidum	r_s_ = 0,58; <0.0001 r_s_= −0.64; <0.0001	Cerebellum Pallidum	* b = 12.72; <0.0001 (and sex; and SPMS-PPMS) * b = −0.39; <0.0001 (and age)

CGMV: cortical gray matter volume; WMV: white matter volume; EDSS: expanded disability status cale; PST: processing speed test. r_s_: Spearman rank correlation coefficient. * *p* < 0.05.

## Data Availability

The data presented in this study are available on request from the corresponding author.
